# Linking Leader’s Behavioral Integrity With Workplace Ostracism: A Mediated-Moderated Model

**DOI:** 10.3389/fpsyg.2022.726009

**Published:** 2022-06-20

**Authors:** Seemab Chaman, Sadia Shaheen, Asrar Hussain

**Affiliations:** ^1^Department of Business Administration, University of Kotli Azad Jammu and Kashmir, Kotli, Pakistan; ^2^Lyallpur Business School, Government College University Faisalabad, Faisalabad, Pakistan; ^3^Department of Management Sciences, Capital University of Science & Technology, Islamabad, Pakistan

**Keywords:** leader’s behavioral integrity, interpersonal trust, narcissistic personality, workplace ostracism, social exchange theory

## Abstract

Drawing on the social exchange theory (SET) and research on leadership influences, we developed and inspected a multilevel model to test the conditions and mechanisms through which a leader’s behavioral integrity (LBI) deters workplace ostracism (WO). We used trust as a mediator and the narcissistic personality of a leader as a boundary condition in the connection between a LBI and WO. Data were collected from 249 employees working in different five- and four-star hotels in Pakistan over three time lags. The statistical results revealed that a LBI reduces WO. Additionally, a LBI has an indirect effect on WO through interpersonal trust. We did not find statistical support for the moderating role of the narcissistic personality of a leader in the relationship between a LBI and WO. Implications, along with limitations and future research directions, are also discussed.

## Introduction

Recently, research on leaders’ behavioral integrity is gaining much more attention from academic researchers and practitioners (Guohao et al., 2021). A leader’s behavioral integrity (LBI) holds key importance in boosting positive workplace attitudes and avoiding ethical breakdowns (Yip and Walker, 2021). LBI is described as “an alignment between a leader’s words and actions” (Simons, 2002). Several studies suggest leaders’ behavioral integrity results in advancing positive outcomes such as helping behavior (Usman et al., 2021), organizational identification (Ete et al., 2021), occupational safety (Halbesleben et al., 2013), and employee commitment and performance (Leroy et al., 2012). Based on the above-stated studies, it would not be wrong to say that a LBI is a truism for leadership efficacy. LBI gained immense importance due to its undeniable outcomes in the workplace (Vogelgesang et al., 2021). In contempt of its vibrant worth, the construct LBI is rarely investigated with its wide antecedents and outcomes (Choi et al., 2020; Ete et al., 2021; Vogelgesang et al., 2021). Therefore, several researchers call for further investigations of leaders’ behavioral integrity (Ete et al., 2021; Javed et al., 2021). The LBI has the potential to curb negative workplace attitudes and behaviors (Choi et al., 2020). Thus, we suggested workplace ostracism (WO) can be minimized by using a LBI as a social tool.

WO refers to a situation in which employees feel that they are ignored by their coworkers as well as their supervisors (Ferris et al., 2008b). WO affects the social belongings of employees, which in turn affects their social needs (Ferris et al., 2012; Wu et al., 2021). So, employees become unable to fulfill their social and psychological needs, which in turn affects them mentally and physically (Singh and Srivastava, 2021). Guzzo and Dickson (1996) reported in their study that working in a team contributes toward individuals and group effectiveness, which shows the importance of social interactions and group cohesiveness. However, WO reduces social interactions and, in turn, reduces the level of employee contributions to the workplace (Gürlek, 2021). The employees working in different organizations become ostracized, which may lower their performance (Ferris et al., 2008b; Lustenberger and Jagacinski, 2010; Choi, 2020; Al-Atwi et al., 2021). Several researchers (Hauge et al., 2010; Wu et al., 2011; Al-Atwi et al., 2021) have studied the influence of WO and its outcomes in the workplace. For example, employees who become ostracized may show unfavorable job attitudes, such as lower job performance (Leung et al., 2011; Al-Atwi et al., 2021), high turnover intentions (Singh and Srivastava, 2021), and even engage in counterproductive workplace behavior (Zhao et al., 2013; Jahanzeb and Fatima, 2018).

There are several studies on the outcomes of WO but only a few studies on the antecedents of ostracism (Al-Atwi et al., 2021). Leadership is a fundamental but overlooked environmental stimuli that have the potential to deal with ostracism and to stimulate a shared way of thinking and a way of boosting interpersonal relationships (Kanwal et al., 2019). Leaders formulate a culture of mutual relationships, support, and inclusion (Ali et al., 2020). Researchers also suggest that practitioners can use leadership as a tool to eliminate, reduce, and handle ostracism (Brown et al., 2005). Drawing from the social exchange theory (SET) (Blau, 1964; Cropanzano and Mitchell, 2005), the extent to which individuals trust their leader, the more bonds between leader and follower develop, which eventually lead toward alleviating ostracism. Employees express confidence toward their leader when he is practicing integrity through his words and actions (Maqsood and Ahm Shamsuzzoha, 2018). Palanski and Yammarino (2009) also suggested that a LBI boosts employee trust in the leader.

The narcissistic personality of an individual also affects ostracism in the workplace (Campbell et al., 2011). It can be characterized as magnificence, superiority, egocentricity, prerogative, and insubstantial self-esteem (Rosenthal and Pittinsky, 2006). According to Penney and Spector (2002), the narcissistic personality of a leader influences their subordinates to involve in negative behavior in the workplace. In organizational settings, a leader with a narcissistic personality involves themselves in negative activities, shows aggression toward their subordinates, neglects their ideas and suggestions, and excludes (ostracizes) employees in organizational activities (Bushman et al., 1998; Pulich and Tourigny, 2004; Asrar-ul-Haq and Anjum, 2020). In line with conservation of resources theory (Hobfoll et al., 2018), when employees interact with a narcissistic leader, it exhausts their psychological resources. Consequently, depletion of resources and a fear of the narcissistic leader weaken his bond with the leader.

The objective of this study was to investigate the connection among a LBI and WO in the service industry of Pakistan. This research also intent to inspect the mediating role of interpersonal trust and the moderating role of narcissistic personality in the connection between LBI and WO. The study has several contributions to the available literature. First, this study tries to answer the recent calls for research on the investigation of antecedents as well as different leadership styles in minimizing WO (Kanwal et al., 2019; Sarwar et al., 2020). Second, we also investigated how individual, organizational, and interpersonal differences affect WO. Third, there is an immense need to investigate these factors to overcome the destructive upshot of WO in service organizations in collectivist societies, where it has severe consequences. Researchers suggest the services sector has been considered a highly labor-intensive sector where interaction between customers and employers takes place frequently. Thus, the services sector is highly dependent on its employees for the proper functioning of the organization. Hence, adding a contextual contribution to provide empirical investigation on how to taper off WO in Asian communities, whereas most of the research on ostracism has been conducted in western communities. Fourth, the development of an integrative model of WO will add great significance to the existing literature.

## Theoretical Background and Hypotheses Development

### Leader’s Behavioral Integrity and Workplace Ostracism

In the current research, several researchers identified the numerous antecedents of WO. Such as to Christensen-Salem et al. (2021), suggest ethical leadership deters both group and individual-level ostracism. Similarly, Kanwal et al. (2019) found several leadership styles as antecedents of ostracism, such as Laissez-faire leadership, transformational leadership, authoritative leadership, and transactional leadership style. Relying on the above-stated studies, the importance of leadership styles in deterring WO cannot be ignored. The available literature on leadership styles also suggest that the LBI have the potential to minimize several negative workplace behaviors, such as deviant workplace behavior (Dineen et al., 2006). Thus, we considered LBI as an antecedent of WO.

Simons (2002) defined leader behavioral integrity as “the perceived pattern of alignment between the leader’s words and deeds or, in other words, the extent that leaders are seen as practicing what they preach.” From time to time, supervisors adopt a certain set of norms and values, but sometimes their actions lack these newly embedded sets of values, which reflect a breach of trust of the followers (Ishaq et al., 2021). This misalignment of words and actions is generally the result of a target achievement craze or requirement, which negatively impacts their subordinates and the work environment. In this capitalist paradigm, it is necessary for organizations to use minimum resources and generate maximum outputs (Becker and Gerhart, 1996). Managers and supervisors have to accomplish these specific goals and lead their teams or groups (Arshad et al., 2021). Moreover, this clash between values and actions results in misconceptions and causes dissatisfaction, peer conflicts, and bad management (Vogelgesang et al., 2020). On the contrary, a LBI boosts trust between followers, and, due to this reason, they are more motivated to perform better and avoid destructive behaviors in the workplace (Yang and Wei, 2018; Choi et al., 2020; Ete et al., 2021).

Ete et al. (2021) found a LBI results in causing certainty among followers. Due to having these feelings of certainty regarding the leaders’ endeavors, the subordinates are more likely to trust in the leader. Additionally, according to belongingness theory, leaders who are highly capricious are less reliable than employees, who prefer to maintain a distance from such types of leaders (Choi et al., 2020). Relying on the SET (Blau, 1964), we postulated that when a leader is highly unreliable and there is no consistency between his actions and words, the mutual relationship between leader and follower will be in danger, and followers will see him as untrustworthy or unpredictable (Dirks and Ferrin, 2002). The discrepancy between the words and actions of the leader disrupts the beliefs of the followers, and the followers begin to assume that the leader is not genuinely concerned about the followers’ wellbeing (Palanski and Yammarino, 2011). Thus, their beliefs jeopardize the trust between leader and follower.

If the leader and follower have a trusting relationship, then they can spontaneously share happiness and sorrows with each other (Lapidot et al., 2007; Carnevale et al., 2020). Additionally, the element of trust encourages followers to reciprocate in a positive manner with the leader.

Once followers perceive their leader as untrustworthy, they avoid interaction with the leader, are not willing to share their ideas, and make themselves susceptible to those who have to contravene their trust (Lapidot et al., 2007; Lacroix and Pircher Verdorfer, 2017). Thus, the discernment of mistrust is considered a threat to an individual, group, and organizational functioning; wellbeing; and social exchange (Blau, 2017). Consequently, all these factors translate into WO. Thus, in the light of evidence, it is hypothesized as follows:

***H1**: LBI is negatively related to WO.*

### Interpersonal Trust as a Mediator

McAllister (1995) referred to “interpersonal trust as the extent to which a person is confident in and willing to act on the basis of the words, actions, and decisions of another.” The conceptualization of the interpersonal trust phenomenon varies. Literature provides an immense amount of evidence regarding the multi-range of interpersonal trust. From the perspective of leader and employee, researchers consider the phenomenon of interpersonal trust as trust in supervisor and define it as “the willingness of one individual to be vulnerable to another” (Ferrin and Lyu, 2018; Ete et al., 2021). Trust helps in building a positive work atmosphere in the organization on the idea of honesty, morally supportive, and helpful relationships. It permits idea sharing without hesitation and hurdles, which affect information quantity and quality (Klaussner, 2012). It helps to motivate employees to enthusiastically cooperate and support each other in handling work-related issues (Saleem et al., 2020). The workplace environment has a valuable impact on employees, and a trust-based work environment helps in character building and gives the philosophy of honesty and moral support-based relationship (Lapidot et al., 2007). In organizations in which trust prevails, effective communication is done without hesitation, employees share their ideas and the problems they are encountering with their immediate bosses. SET (Blau, 2017) argues that positive interactions among individuals are based on the mutual response, in leader and follower case, it exists when both leader and followers’ words, action, and deeds are on the same page (Simons et al., 2007). Researchers and psychologists use SET (Blau, 2017) as a tool to understand how fairness and justice nourish the development of interpersonal trust and several researchers follow the same pattern (Deluga, 1994; Aryee et al., 2002; Camerman et al., 2007; Schoorman et al., 2007). Employee’s trust in their leaders results from their leaders’ behavioral integrity (Palanski and Yammarino, 2009). In an organizational setting, in order to achieve common organizational goals, individuals are supposed to work with coordination, but it is terrible with the existence of ostracism at the workplace (Robinson, 1996; Kramer, 1999). Thus, the presence of behavioral integrity is necessary to create the interpersonal trust that is needed for establishing coordination between workers. Zhang and Dai (2015) reported that interpersonal trust has a noteworthy toxic relationship with ostracism, which means in organizations where interpersonal trust exists among leader and employee, which channels through alignment of their action, words, and deeds, have a strong work environment and ostracism is reduced. Thus, on the basis of the above literature, it is predicted as follows:

***H2:** Interpersonal trust mediates the relationship between a LBI and WO.*

### Narcissistic Personality as a Moderator

Narcissism is recognized as a personality trait incorporating magnificence, superiority, egocentricity, prerogative, insubstantial dignity, and aggression (Rosenthal and Pittinsky, 2006). For the past two decades, this particular concept has been attaining importance in the literature; therefore, psychologists and clinical experts are analyzing this personality trait in multiple dimensions (Asrar-ul-Haq and Anjum, 2020; Bernerth, 2020; Fehn and Schütz, 2020). From an organizational perspective, this concept is linked with the emotional stability and instability of individuals related to their performance (Ouimet, 2010). Furthermore, in this perspective of leadership, a leader’s personality influences its followers’ behaviors (Nassif et al., 2020), and it has been observed that the narcissistic personality of a leader enhances the deviant behaviors of the employees (Penney and Spector, 2002; Judge et al., 2006; Nevicka et al., 2018; Zhang et al., 2018). Rosenthal and Pittinsky (2006) characterized the narcissistic personality of an individual as magnificence, superiority, egocentricity, prerogative, and insubstantial self-esteem. In the organizational setting, a leader with a narcissistic personality involves themselves in negative activities, shows aggression toward their subordinates, neglects their ideas and suggestions, and excludes (ostracizes) employees in an organizational setting on the basis of their dislikes, and this sort of behavior is a threat to the employee’s wellbeing (Nevicka et al., 2018; Zhang et al., 2018). Tepper et al. (2006, 2009) conducted a study on the narcissistic personality of leaders and their impact on employees and reported that narcissistic supervisors are emotionally nuclear and are abusive in supervision. Narcissistic leaders are hypersensitive, arrogant, and self-centered (Rosenthal and Pittinsky, 2006). The characteristics of narcissistic leaders create difficulties in interpersonal relationships. The absence of empathy, support, and relatedness drains the employee’s psychological resources (Westman et al., 2004). Relying on the conservation of resources theory (Hobfoll, 2002), a narcissistic leader drains employees’ psychological resources, which shapes their less supportive workplace behaviors, such as ostracism. In consonance with SET (Blau, 1964), a leader’s behavior shapes followers’ reactions through the social exchange process. When employees find their leader as supportive and kind in interpersonal relationships, they return the leader’s favor (Erkutlu and Chafra, 2017). On the contrary, when they find their leader arrogant and less supportive, they reciprocate in the same manner (Blau, 1964). The followers working under narcissistic leaders are more probably to be victims of ill-treatment like WO. Thus, a leader’s narcissistic personality boosts WO and neutralizes the effect of the LBI.

**FIGURE 1:**
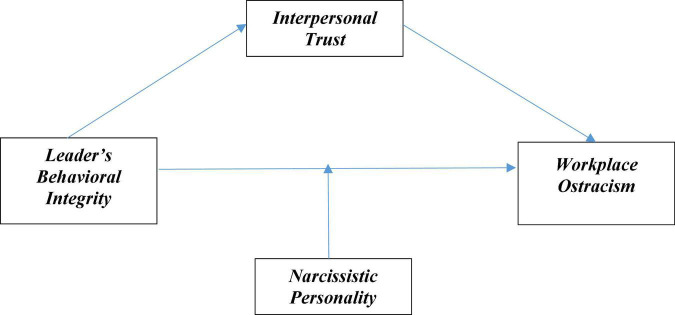
Conceptual framework.

Therefore, leaders will have optimum levels of WO even if they have a high level of LBI due to narcissistic behaviors (i.e., over-confidence, egoism, entitlement, fragile self-possession, and aggression). Therefore, it is expected that WO is affected by a leader’s narcissistic personality, and it will also affect the benefits of behavioral integrity. Therefore, it is proposed as follows:

***H3:** Narcissistic personality moderates the relationship between a LBI and WO such that the relationship will be stronger when narcissistic personality is high than low narcissistic personality. See Figure 1.*

## Methodology

Data for this study were collected from the service sector of Pakistan. We used a time-lag approach to data collection. We chose five- and four-star hotels in Pakistan from the province of Punjab, Pakistan. Maximum hotels were approached for data collection. But only 39 hotels agreed to participate in the study. After meeting with the managers or heads of the hotels, the purpose of the study was elaborated. It was assured to them that the obtained data will be used only for academic purposes and that it would be used in an aggregate manner. Additionally, your answers will not be considered as correct or incorrect, so be comfortable while filling out the questionnaire. After taking permission from the management and heads of the hotel, the questionnaire was distributed. We collected the data from two sources, namely, the supervisor and the follower. The followers/subordinates responded to the independent variable (the LBI), the mediator (interpersonal trust), and the outcome variable (WO). The supervisors responded with narcissistic personalities. Data were collected from different subsections of hotels, such as marketing, security, housekeeping, accounts, maintenance, and human resource.

Responses were gathered on three time lags by applying a 1-month time interval between each time lag. In time period 1, responses were gathered on the LBI. In time period 2, responses were gathered on narcissistic personality and interpersonal trust. In time period 3, responses were gathered on WO. To reduce the possible issue of common method bias, we applied temporal separation between variables, which is suggested by several researchers (Podsakoff et al., 2003; Shaheen et al., 2017; Khan et al., 2020; Naseer et al., 2020). We used a convenience sampling technique to collect the data. The nesting issue has been controlled in the hypothesis analysis by recruiting one employee-leader dyad from every team. A unique identification number was assigned to each participant so that the responses at different time lags could be matched. The unique identification number also helped us in matching each leader’s response with each employee’s response.

A total of 360 questionnaires were distributed. In time period 1, we received 326 completely filled questionnaires, having a response rate of 90.50%. In time period 2, we contacted only those members who completed the survey in time period 1 and a total of 326 were floated. We received 299 completely filled questionnaires with a response rate of 91.71%. In time period 3, a total of 299 surveys were distributed, and we received 249 completely filled questionnaires, having a response rate of 83.27%.

According to the demographic information, 78.3% were male and 21.7% were female. With respect to age, 21.1% belongs to the age category of 20–30 years, 69.9% belongs to the age category of 31–40 years, and 9% belongs to the age group of 40 years and above. Regarding employee tenure, 6% had 1–3 years of experience, 13.3% had 4–6 years of experience, 37.7% had 7–9 years of experience, and 43% had 10 and above years of experience. Related to education, 24.5% belong to below 14 years of education, 19.7% had 14 years of education, 43% had 16 years of education, and 12.8% had a diploma and any other education.

### Control Variables

Several researchers used demographic variables as control variables in ostracism studies. Researchers suggest that feelings of ostracism might have different effects for employees who have different age groups and gender, along with having different ranks of education and experience (Ferris et al., 2008a; Jahanzeb and Fatima, 2018). Accordingly, these different levels may also affect interpersonal trust (Kannan-Narasimhan and Lawrence, 2012). Therefore, we controlled for gender, age, education, and experience in the regression analysis. Demographic variables were measured by using coding. We measured gender, age, education, and experience as ordinal variables by using different categories. Such as, gender was measured in two groups, namely, male (classify as 1) and female (classify as 2). Similarly, age was also measured as an ordinal variable by using five categories, such as 20–24 years of age was classify as 1, 25–29 years of age was classify as 2, 30–34 years of age was classify as 3, 35–39 years of age was classify as 4, and 40 and above were classify as 5. The same procedure was used to measure other demographic variables.

### Measures

All the items in the questionnaire were responded to by using a 5-point Likert scale from 1 [strongly disagree (SD)] to 5 [strongly agree (SA)].

### Leader’s Behavioral Integrity

Employees’ perceptions of their LBI were measured using the 8-item scale developed by Simons et al. (2007). Sample questions include “There is a match between my manager’s words and actions” and “My manager conducts himself/herself by the same values he/she talks about.” The Cronbach’s α was 0.91.

### Interpersonal Trust

The 11-item scale developed by McAllister (1995) was used to measure interpersonal trust. This measure includes 5 items for affect-based trust and 6 items for cognition-based trust. The sample items include “My leader and I have a sharing relationship,” “We can both freely share our ideas, feelings, and hopes,” and “My leader approaches his/her job with professionalism and dedication.” The Cronbach’s α was 0.91.

### Narcissistic Personality

The Narcissistic Personality Inventory-16 developed by Ames et al. (2006) was used to assess the narcissistic personality of the leader. This is a 16-item scale. The sample includes “I know that I am good because everybody keeps telling me so,” “I like to be the center of attention,” and “I think I am a special person.” The Cronbach’s α was 0.86.

### Workplace Ostracism

A 10-item scale developed by Ferris et al. (2008a) was used to measure WO. Sample items include “Others ignored you at work,” “Your greetings have gone unanswered at work,” and “Others left the area when you entered.” The Cronbach’s α was 0.93.

## Results

### Measurement Model

Before testing the main hypothesis, we analyzed the measurement model through confirmatory factor analysis (CFA) to determine the discriminant validity. We performed CFA by using AMOS software, which has been widely used by researchers (Naseer et al., 2020; Irshad et al., 2021). The obtained results support the uniqueness of all the theoretical variables as additionally also prove that the hypothesized model is free from common method bias. According to the statistical results, the four-factor model had an acceptable model fit in contrast to alternative models such as the three-factor and two-factor models. χ^2^/df = 1.455, RMSEA = 0.043, IFI = 0.95, TLI = 0.94, and CFI = 0.95 (Hair et al., 2006). See Table 1.

**TABLE 1:** Fit indices of hypothesized model and alternative models.

Model	χ^2^	df	Δχ^2^	Δ *df*	RMSEA	CFI	SRMR
M0-Hypothesized 4-Factor Model	831.024	571	–	–	0.043	0.95	0.0516
M1-3-Factor Model by combining LBI and IT	1888.535	591	1057.511	20	094	0.754	0.1128
M2-2-Factor Model by combining LBI, IT, and NP	2969.825	593	2138.80	22	0.127	0.549	0.1590

*LBI, leader’s behavioral integrity; IT, interpersonal trust; NP, narcissistic personality; WOS, workplace ostracism.*

### Descriptive Analysis, Correlation Analysis, Average Variance Extracted, and Square Root of Average Variance Extracted

In Table 2, mean, standard deviation, correlation analysis, average variance extracted (AVE), and the square root of average variance extracted (SQRAVE) have been reported. The construct validity of the theoretical model was assessed through convergent and construct validity using SPSS 23. The convergent validity is satisfactory as depicted by AVE values. The values of AVE are above 0.5, which is a cutoff point recommended by Hair et al. (2006). Additionally, the discriminant validity was also assessed by utilizing a criterion recommended by Fornell and Larcker (1981). The statistical results prove the discriminant validity of the theoretical model because the square root values of all the theoretical variables are bigger than their correlations (Fornell and Larcker, 1981) (refer to Table 2 the square root values given on diagonal). The Cronbach’s alpha (α) reliabilities of LBI, interpersonal trust, narcissistic personality, and WO were 0.91, 0.91, 0.68, and 0.93, respectively, which also satisfied the cutoff criteria, which is 0.6 (Hair et al., 2006, Hair et al., 2016). The correlation analysis is also presented in Table 2.

**TABLE 2:** Descriptive statistics, correlation analysis, and discriminant validity.

	Variables	Mean	SD	CR	α	AVE	1	2	3	4	5	6	7	8
1	Gender	1.22	0.41				−							
2	Age	1.86	0.53				0.029	−						
3	Education	2.31	0.93				0.058	−0.190**	−					
4	Experience	3.18	0.88				−0.130*	0.011	−0.230**	−				
5	LBI	3.45	0.84	0.913	0.91	0.601	0.088	−0.178**	–0.093	–0.105	**0.775**			
6	IT	3.82	0.85	0.918	0.91	0.652	0.003	−0.196**	0.010	–0.103	0.184**	**0.808**		
7	NP	3.97	0.79	0.912	0.86	0.421	0.017	–0.056	0.009	–0.083	0.010	–0.031	**0.815**	
8	WOS	2.99	0.89	0.933	0.93	0.664	0.083	–0.069	0.0176**	0.266**	−0.159*	−0.290**	–0.106	**0**.**649**

N = 249, *p < 0.05, **P < 0.001. LBI, leader’s behavioral integrity; IT, interpersonal trust; NP, narcissistic personality; WOS, workplace ostracism.

**FIGURE 2:**
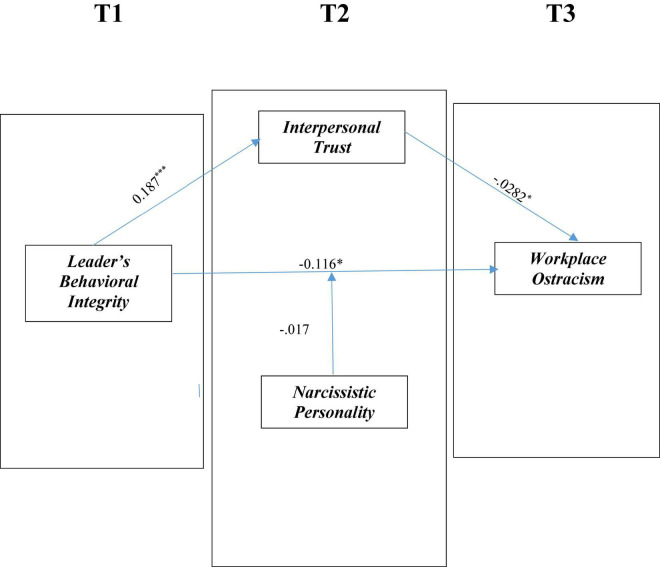
Summary of the findings. T1, time lag 1; T2, time lag 2; T3, time lag 3; *p < 0.10, ***p < 0.01.

The correlation analysis shows leaders’ behavioral integrity is positively and significantly related with the interpersonal trust (*r* = 0.184, *P* < 0.0), positively but insignificantly related with the narcissistic personality (*r* = 0.10, *P* > 0.05), and negatively but significantly related with the WO (*r* = –0.159, *P* < 0.01). Likewise, interpersonal trust is negatively and insignificantly related with the narcissistic personality (*r* = –0.031, *P* > 0.05) and negatively but significantly related with the WO (*r* = –0.290, *P* < 0.01). Narcissistic personality is negatively and insignificantly related with the WO (*r* = –0.106, *P* > 0.05). Please see Figure 2.

### Hypothesis Analysis

We utilized the most popular method proposed by Preacher et al. (2007) to analyze the proposed model. Specifically, the hypotheses were confirmed using PROCESS macro by Hayes (2013). We utilized model 4 to test the mediation hypothesis and model 3 to test the moderation hypothesis, which have been widely used previously by academic researchers to test mediation and moderation relationships. We controlled the demographic variables in the regression analysis. The findings of the hypothesis analysis are presented in Tables 3, 4. In line with hypothesis 1, LBI is negatively related with the WO, which was proven by the obtained results (*r* = –0.116, *P* < 0.01). Thus, hypothesis 1 is supported. Hypothesis 2 stated that interpersonal trust mediates the connection between LBI and WO, which was also supported by the results. As 95% of bootstrapped confidence interval results did not have zero [–0.100, –0.017] and based on statistical results, it is stated that interpersonal trust acts as a mediator in the direct connection between LBI and WO. Hypothesis 2 is supported. Hypothesis 3 stated that narcissistic personality moderates the relationship between LBI and WO such that the connection will be weaker when narcissistic personality is high. We did not find support for this hypothesis as proven by the statistical results of the interaction term (*r* = –0.017, *P* > 0.05). For this reason, hypothesis 3 is rejected.

**TABLE 3:** Results of the mediation analyses.

	Coefficient	SE	Bootstrap 95% CI
*IV to mediator (A path)*			
LBI → IT	0.187***	0.0635	
*Mediator to DV (B path)*			
IT → WOS	−0.0282*	0.064	
*Total effect of IV on DV (C path)*	−0.169*	0.067	
*Direct effect of IV on DV (C path)*	−0.116*	0.065	
*An indirect effect of IV on DV through the proposed mediator*			
LBI → IT → WOS	−0.052*	0.021	[−0.100, −0.017]

N = 249, *p < 0.10, ***p < 0.01. LBI, leader’s behavioral integrity; IT, interpersonal trust; NP, narcissistic personality; WOS, workplace ostracism.

**TABLE 4:** Moderation analysis.

Moderator: Narcissistic personality	Dependent variable: Workplace ostracism			
	β	SE	LL CL	UL CL
Constant	3.80	1.29	1.246	6.364
*Leader’s behavioral*	–0.100	0.35	–0.8025	0.600
*Narcissistic personality*	–0.057	0.323	–0.694	0.578
*Leader’s behavioral* × *Narcissistic personality*	–0.017	0.088	–0.191	0.157

## Discussion

WO has been considered a staid threat to employees’ workplace behaviors and attitudes as verified by several researchers (Sarwar et al., 2020; Takhsha et al., 2020; Singh and Srivastava, 2021; Uslu, 2021). Due to its negative effects, it can also harm the wellbeing of the service sector and its employees in delivering effective services. Thus, it is pivotal to understand how to minimize ostracism, particularly in service organizations. The results of this study address this central issue. The findings of the study reveal a LBI, such as an alignment between a leader’s words and actions, fair treatment of the leader, and morality of the leader, have a negative impact on WO. Previous findings on the leader-member exchange, ethical leadership, transformational leadership, spiritual leadership, and WO also provide support for our findings (Fiset and Boies, 2018; Babalola et al., 2019; Kanwal et al., 2019; Greenbuam et al., 2020). Additionally, SET (Blau, 2017) serves as a theoretical pillar in justifying the obtained results. In line with SET (Blau, 2017), helpful behaviors are reciprocated with positive acts. When employees experience justice and fair treatment by their leader, a sense of trust is developed. These feelings of trust motivate employees to exchange work-related problems with the leader. Thus, a friendly relationship boosts an environment of collaboration and sharing. Employees feel like a part of the organization. Resultantly, they feel less ostracized. In contrast to a situation where employees do not trust their leader due to his/her misalignment in deeds and acts. In this situation, employees prefer to withhold information and keep their distance from the leader, which ultimately results in WO.

The findings suggest that interpersonal trust mediates the connection between LBI and WO. When employees experience fair treatment by the leader, transparency in words and actions strengthens their trust in their leader (Blau, 2017). The positive feelings regarding a leader’s transparent behavior eventually enhance a follower’s trust, and the aura of trust motivates followers to share their problems with their leader. Contrary to our expectations and available literature, we found no support for the moderating role of the narcissistic personality of the leader in the connection between the LBI and WO. There may be certain reasons for the rejection of this prediction. Pakistan, for example, is categorized as having a high collectivistic culture. Due to the attributes of collectivist culture, leaders wish to maintain harmonious relationships and esteem in the eyes of their followers. Therefore, they avoid demonstrating aggression to their followers. The other reason could be the service sector, where leaders have to play the role as role models in behaving well and having a harmonious relationship with their followers. Therefore, they avoid demonstrating aggression and antipathy toward the followers.

### Theoretical Implications

This study is an extension of the available literature by providing a unique investigation between LBI and WO with the lens of interpersonal trust. Additionally, the investigation of the moderating role of narcissistic personality enriches the literature on leadership and individual differences. Despite having the utmost importance of these constructs in organizational studies, little was known about the impact of LBI on reducing WO in the service sector of Pakistan. Thus, the current study adds an essential contextual contribution to the available literature. Despite having several consequences of WO in the services sector, such as its negative impact on service sector performance, wellbeing, and productivity, the empirical investigation of a LBI in attenuating its harmful impact in the service sector was less investigated.

Additionally, existing empirical pieces of evidence on the negative connection among leadership approaches like ethical leadership, transformational leadership, and spiritual leadership with WO call for further leadership styles to attenuate its destructive impact in the services sector (Kanwal et al., 2019). The empirical investigation of the negative connection between LBI and WO strengthens the literature of leadership in understanding the importance of LBI to overcome ostracism in the service industry.

Additionally, the present literature undermines the empirical shreds of evidence in understanding the role of interpersonal trust as a mediator in the connection between LBI and WO. By establishing interpersonal trust as a mediator in the relationship between a LBI and WO, this study extends the scope of SET theory in understanding the trust as a fundamental source in curbing ostracism. Thus, the empirical investigation adds the potential antecedents of ostracism in the service sector. The outcomes of the research also reinforce the conclusions of the previous investigations that suggested a leader’s positive behavior reduces the negative behavior of employees. The relationship between the variables is mainly theorized based on SET. The SET theory helps us in analyzing the leadership–ostracism relationship. The negative relationship between LBI and ostracism extends the scope of SET in the context of the service sector. Additionally, the inclusion of trust as a mediator in the LBI and ostracism relationship enriches the scope of LBI in reducing ostracism. Finally, the empirical investigation of the moderating role of the narcissistic personality of a leader in strengthening ostracism in the workplace extends the literature of leadership in understanding the negative outcomes of leadership styles in the service sector.

### Practical Implications

We suggested several implications for the practitioners and managers in the services sector to curb the practices of WO. The findings of the study revealed that service organizations have to bear a high cost when employees and their leaders experience ostracism. Therefore, we suggested that it is necessary to deal with the WO before its emergence in the service organization.

We suggested that service organizations should direct their leaders or managers to inspire their followers/employees by developing transparency in their words and actions (behavioral integrity) and provide support so they can develop feelings of relatedness and belongingness, which may help followers mitigate the feelings of WO. To address this concern, organizations need to educate their leaders about the prominence of behavioral integrity in minimizing the impact of WO through training, seminars, and workshops. Leaders should cultivate an atmosphere of trust through their actions and words. For this, leaders need to enforce integrity on a permanent basis. Leaders/managers should create an atmosphere of transparent relationships where employees perceive feelings of trust and relatedness, which can help in safeguarding employees’ social and emotional needs. For this, they should demonstrate a transparent system in all aspects, not only in reward allocation but also in punishments. Such relational transparency helps employees in boosting feelings of trust in their leaders. Resultantly, they will start to rely on their leader without any hesitation, which ultimately results in reducing WO. The interdependence between leader and employee is a robust tool to develop a strong bond between leader and employee. The managers should also consider the consequences of the narcissistic personality of leader (e.g., arrogance, self-centeredness, and low leader member exchange) and take preventive measures to minimize such behaviors through professional trainings and workshops. When the negative impact of the narcissistic personality of leader is weakened, there are greater chances of strengthening the positive impact of LBI on WO.

### Limitations and Future Research Directions

Despite having several strengths, the study is not limitation-free. First, the only time-lag approach is not sufficient to mitigate common method bias. We suggested that a longitudinal approach should be utilized. Second, we emphasized the personality trait of a leader as a moderating mechanism, but it would be very interesting to investigate some cultural variables as a moderating mechanism impacting WO. The findings of the study suggest that LBI relieves ostracism. Furthermore, this line of research could be extended by adding mechanism that helps the practitioners in reducing ostracism in the service sector, such as social support, psychological capital, and ethical leadership. The presence of ostracism has greater threats for collectivist cultures as contrast to individualistic cultures. Collectivist cultures pay more attention to interpersonal relationships, bonding, and pleasant relationships. Thus, employees, leaders, and consumers belonging to collectivist cultures are prominently overwhelmed by ostracism. The study has been conducted in Pakistan, so the findings may not be applicable to individualistic cultures. In the present research we investigated only the antecedents of ostracism in the service organization, which depicts only a half picture of the story; it would be fruitful to analyze the outcomes of ostracism. For example, the behaviors of employees when they experience ostracism (e.g., work engagement, negligence and tardiness, and passion at work).

Third, we considered only the services sector for the investigation of the theoretical model. Furthermore, we suggested a comparison study between services and production organizations to understand the impact of WO.

## Data Availability Statement

The raw data supporting the conclusions of this article will be made available by the authors, without undue reservation.

## Author Contributions

All authors listed have made a substantial, direct, and intellectual contribution to the work, and approved it for publication.

## Conflict of Interest

The authors declare that the research was conducted in the absence of any commercial or financial relationships that could be construed as a potential conflict of interest.

## Publisher’s Note

All claims expressed in this article are solely those of the authors and do not necessarily represent those of their affiliated organizations, or those of the publisher, the editors and the reviewers. Any product that may be evaluated in this article, or claim that may be made by its manufacturer, is not guaranteed or endorsed by the publisher.
